# Application of fourier transform and proteochemometrics principles to protein engineering

**DOI:** 10.1186/s12859-018-2407-8

**Published:** 2018-10-16

**Authors:** Frédéric Cadet, Nicolas Fontaine, Iyanar Vetrivel, Matthieu Ng Fuk Chong, Olivier Savriama, Xavier Cadet, Philippe Charton

**Affiliations:** Peaccel SAS, Protein Engineering ACCELerator, n°6 Square Albin Cachot, Box 42, 75013 Paris, France

**Keywords:** Directed evolution, Protein sequence activity relationship, Protein spectrum, Rational screening, Statistical modelling

## Abstract

**Background:**

Connecting the dots between the protein sequence and its function is of fundamental interest for protein engineers. In-silico methods are useful in this quest especially when structural information is not available. In this study we propose a mutant library screening tool called iSAR (innovative Sequence Activity Relationship) that relies on the physicochemical properties of the amino acids, digital signal processing and partial least squares regression to uncover these sequence-function correlations.

**Results:**

We show that the digitalized representation of the protein sequence in the form of a Fourier spectrum can be used as an efficient descriptor to model the sequence-activity relationship of proteins. The iSAR methodology that we have developed identifies high fitness mutants from mutant libraries relying on physicochemical properties of the amino acids, digital signal processing and regression techniques. iSAR correlates variations caused by mutations in spectra with biological activity/fitness. It takes into account the impact of mutations on the whole spectrum and does not focus on local fitness alone. The utility of the method is illustrated on 4 datasets: cytochrome P450 for thermostability, TNF-alpha for binding affinity, GLP-2 for potency and enterotoxins for thermostability. The choice of the datasets has been made such as to illustrate the ability of the method to perform when limited training data is available and also when novel mutations appear in the test set, that have not been featured in the training set.

**Conclusion:**

The combination of Fast Fourier Transform and Partial Least Squares regression is efficient in capturing the effects of mutations on the function of the protein. iSAR is a fast algorithm which can be implemented with limited computational resources and can make effective predictions even if the training set is limited in size.

**Electronic supplementary material:**

The online version of this article (10.1186/s12859-018-2407-8) contains supplementary material, which is available to authorized users.

## Background

Humans have exploited biological systems to their advantage since the dawn of civilization for example domestication of animals and crop cultivation, but it is not until the second half of the twentieth century that we have extended the sophistication to the molecular level. More specifically this involves engineering proteins to perform novel bio-processes or improve their efficiency or induce them to function in unnatural conditions or a combination of the above. Early efforts involved introducing mutations randomly to the protein primary sequence and then screening for the ones with desired quality [1], later site directed mutagenesis enabled the modification of specific residues [2]. With the advent of the more recent CRISPR/Cas9 technology [3], the genome itself can be edited to produce a protein of desired interest with the context of a living cell. Hence it becomes necessary to draw a correlation between an artificially introduced change to the protein and the effect it has on its characteristics [4–7].

One of the approaches that uses in-silico methods to decipher these correlations consists of converting the primary structure of the protein into a string of values corresponding to the physicochemical properties of the amino acids. The AAindex is a database that holds more than 500 such physicochemical properties for the 20 standard amino acids and correlation between these indices are also listed [8, 9]. When this study was conducted 544 indices were described, currently the database holds 566 indices. Veljković et al. exploited one such index, the electron-ion interaction potential (EIIP) [10] to find the relationships between biological sequences and their functions using digital signal processing [11]. The closely related method called Resonant Recognition Model (RRM) [12] uses Discrete Fourier Transform (DFT) to analyse the signals and attempts to correlate them with function. RRM has been applied to a variety of studies ranging from the electromagnetic nature of biomolecular interactions [13] to predicting ‘hot spots’ in the hormone prolactin [14] and more recently for the analysis of tumour necrosis factor [15], however this list is far from comprehensive.

Previous attempts have been made to study the effect of amino acid substitutions on the activity, function and stability of proteins whose structures have been resolved. This resulted in Quantitative Structure Function Relationship (QSFR) and Quantitative Structure Stability Relationship (QSSR) studies [16–18]. Particularly, the impact of mutations on the stability of proteins is of specific industrial interest and has been the subject of various studies. These tools have also been made available as web servers [19]. There are also web servers that integrate multiple tools to provide the user with an option to perform a wider gamut of analysis on the mutants of their interest [19, 20]. Although these structure dependent methods are effective in deriving the correlation between the mutation and its effect on the protein activity, they are limited by their requirement of the availability of the protein structure. Hence interest lies in deciphering the impact of mutations irrespective of the availability of structural information, purely based on physicochemical and other molecular properties of the varying amino acids and statistical analysis thereof.

In 2001, Lapinsh et al. [21] termed Proteochemometrics a novel method for the analysis of drug receptor interactions. This method uses descriptors of both the interacting species, i.e. drug and protein receptor [21]. These descriptors can be used independently or combined i.e. only those of the protein receptor or/and of the drug (peptide or small chemical compound). This approach refers to chemometrics applied to proteins. Interactions between amino-acids residues at intra-molecular positions of mutations or mutated protein domains, independently or combined for the interacting species, are taken into account during the modelling. When either the protein receptor or/and peptide is considered, Lapinsh’s approach is a protein engineering method for identifying amino acid residues for variation in a protein variant library in order to affect a desired biological activity/fitness. It is based on a training set of a protein variant library, where the data are sequence information and activity for each protein (or peptide) variant. From the data, a sequence-activity model is developed to predict activity as a function of amino acid residue type and corresponding position in a protein sequence. The mathematical model is a regression model such as a partial least squares model that includes at least one non-linear terms, each representing an interaction between two or more amino acid residues in the protein sequence (or protein domains). The sequence-activity model can distinguish amino acid residues that have a significant impact on the desired activity from those that do not have. The model allows thus to identify one or more amino acid residues at specific positions, that are predicted to impact the activity, for variation to impact (i.e. increase or decrease) the desired activity. A non-linear term is a cross-product term comprising a product of one variable representing the presence of one interacting residue and another variable representing the presence of another interacting residue (or interacting domain). During the modelling, a selection of one or more cross-product terms, from a group of potential cross-product terms, is done in order to select those representing true structural interactions that have a significant impact on the targeted activity. This protein engineering approach has been successfully applied in different cases such as: for the prediction of MSH peptide binding to melanocortin receptors [22], or the prediction of targets for anticancer drugs [23], for the selectivity of serine protease [24] or more recently for the prediction of Peptide Binding to HLA-DP Proteins [25].

QSAR methods applied to modelling peptide or protein activity [26–28] that consist in using sets of descriptors derived from sequence information in essence, implements this approach. This was also known as recently termed as Protein Sequence Activity Relationship or ProSAR [29]. In this last paper one implementation of such methodology is presented and relied on the binary encoding of the amino acid sequences of the wild type and a collection of few mutants whose activities are known. A statistical model is built to represent the relationship between the mutation and the activity [29]. Subsequent mutant libraries are generated by favouring those mutations that positively affect the activity. This methodology has demonstrated to be able to obtain a 4000-fold improvement in the volumetric productivity of the enzyme halohydrin dehalogenase [30]. An evaluation of the methodology was recently described by [31].

**Fig. 1:**
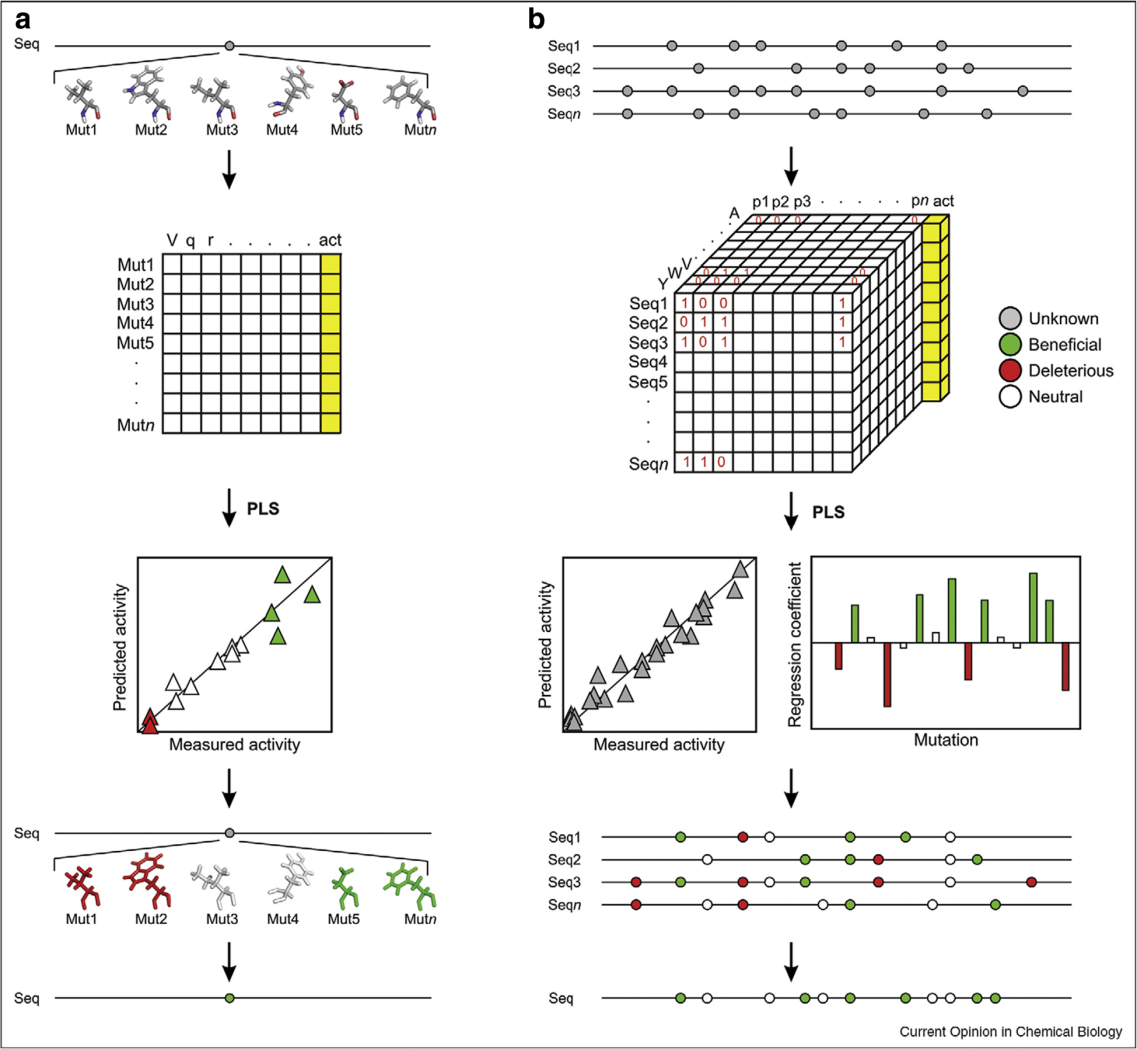
Principles of statistical methods used to model structure or sequence to activity relationship (Damborský and Brezovsky [41] reproduced with permissions). **a** Schema illustrating the principles behind Quantitative Structure to Function Relationship method whereby numerical descriptors derived from structure are regressed on the activity data (yellow column). **b** Principles behind Protein Sequence to Activity Relationship methods whereby numerical descriptors derived from sequence are regressed on the activity data

Both ProSAR and the structure dependant QSFR methodologies, as shown in Fig. 1, fall under the category of iterative mutant screening methods. The main assumption in iterative mutant library screening methods is that the effects of the mutations are additive in nature [32–34]. But this additivity is not absolute and this is reflected in the challenges faced in model building. Experimentally generating single substituted mutant libraries is much easier than combinatorial mutant libraries, especially due to the exponential increase in the number of combinations to explore with the increase in number of mutated positions. Hence this additive nature of the fitness property is exploited to avoid exhaustively searching the vast sequence space.

Regression methods try to establish a regression function that relates independent variables to a dependent variable. Classical regression methods like linear regression and least squares regressions cannot be used to find the regression function in this case because the two assumptions that the sample size is larger than the number of variables and the non-correlation among the independent variables clearly does not hold well. In such cases a regression method called the Partial Least Squares (PLS) method is used to overcome these limitations [35, 36]. Although PLS method was initially developed for application in the economics domain, after 35 years of its development, it has found applications in diverse fields [21, 37–39]. PLS regression is ideally suited for datasets with many collinear variables (linear and interaction terms) and few observations (sequences) [40, 41].

In this work we propose a novel method that combines a digital signal processing technique and PLS regression technique as a predictive tool for the screening of protein mutant libraries. We call this method iSAR for innovative Sequence Activity Relationship. iSAR converts the amino acid sequence into a protein spectrum after its numerical encoding using selected physicochemical properties of its constitutive amino acids and subsequent treatment using Fast Fourier Transform (FFT). It then finds a regression function that correlates changes brought in the spectra due to mutations and observed changes in the fitness of the protein variants as measured experimentally. Here we use “fitness” as generic term to denote a desirable character of a protein like catalytic efficacy, catalytic activity, *K*_*m*_, binding affinity, thermostability, solubility, aggregation, potency etc. Unlike previously developed methods, we do not limit ourselves to a single amino acid physicochemical property but examine all those listed in the AAindex database and choose the one that is most informative. The spectrum that is calculated is the energy spectra obtained after FFT. We have for the first time attempted to use protein spectra for statistical modelling in order to predict the effect of mutations on the fitness of protein variants, i.e. to establish protein sequence to activity relationship. Our method is independent of the availability of structure information, does not confine itself to only the local effects of the mutation and is computationally less demanding. We demonstrate the utility of the method to identify protein variants with better fitness on four experimentally verified datasets.

## Methods

### Experimental datasets

The iSAR methodology requires experimental data to bring to light correlations between mutated sequences and their corresponding fitness. Based on these correlations, statistical models are built which in turn enable us to predict the fitness of novel mutants. We have used four such datasets to demonstrate the robustness of the iSAR methodology. The performance of iSAR on these datasets have been evaluated and cross-validated, the procedure is described below and the results obtained are discussed in the subsequent sections. The datasets chosen were diverse in both the fitness criteria being tested for and the size of the mutant library.

The first dataset involves the potency of 31 alanine variants of the Glucagon like peptide-2 (GLP-2) with respect to the activation of its receptor [42]. GLP-2 is a short 33 residues peptide whose increase in activity has direct implication in the control of epithelial growth in the intestine. The value for the corresponding receptor activation for the 31 alanine variants of GLP-2 is defined as the fold increase over basal cAMP production and are ranged from 0.7 to 10.4. The second dataset concerns the thermostability of 242 chimeric cytochrome P450 sequences [43]. Cytochrome P450 are heme-containing redox enzymes whose T_50_ (temperature at which 50% of the protein denatures after 10 min. of incubation) ranges from 39.2 °C to 64.48 °C. The third dataset is for thermostability as well but for staphylococcal enterotoxins E and A (SEE and SEA) [44]. SEE and SEA are Super-antigens (SAgs) that elicit a strong immune response by activating T-cells. The denaturation temperatures (T_m_) for the 10 mutants + WT SEE + WT SEA ranged from 55.1 °C to 73.3 °C. The fourth dataset from Mukai et al. is a collection of 20 mutants and one WT Tumour Necrosis Factor (TNF) sequences [45]. TNF is an important cytokine that suppresses carcinogenesis and excludes infectious pathogens to maintain homeostasis. The relative affinity (%K_d_) of TNF to its two receptors, TNFR1 and TNFR2 is computed as a single ratio of log_10_(R1/R2) which ranges from 0 to 2.87, where R1 and R2 are affinities of TNF to TNFR1 and TNFR2 respectively as measured by IC_50_ assays in ng/ml. The datasets are summarized in Table 1.

**Table 1:** Characteristics of the experimental datasets. *n* is the number of mutated positions and *k* is the number of residues at each position

Dataset	Size of dataset	*n*	*k*	Theoretical size of sequence space	Length of protein sequence
Cyt P450	242	8	3	6561	464–466
GLP-2	31	31	2	2.147 billion	33
Enterotoxin	12	40	2	1099.5 billion	233
TNF	21	17	[2, 7, 4, 6, 2, 9, 9, 9, 9, 9, 2, 2, 2, 2, 6, 8, 7]	213.3 billion	157

The theoretical size of sequence space *S* is calculated as the product all *k* values for all mutated positions

The list of all variants and their corresponding measured biological activity for all 4 datasets are further detailed in Additional file 1: Tables S1-S4.

### Statistical measures of correlation

We use the coefficient of determination (R^2^) and the Root Mean Squared Error in Cross-Validation (cvRMSE) as quantitative and qualitative measures of correlation between the measured and predicted values of the different fitness criteria. The cvRMSE allows to construct and select the best models. The predictive ability of these models relies in the R^2^ values. While R^2^ is a measure of the extent of agreement between the measured and predicted fitness, cvRMSE represents the extent to which the predictions vary when different training sets are used. R^2^ and cvRMSE are calculated as follows in Eqs. 1 and 2 respectively:

1 $$ {R}^2=\frac{{\left({\sum}_{i=1}^S\left({y}_i-\overline{y}\right)\left({\hat{y}}_i-\overline{\hat{y}}\right)\right)}^2}{\sum \limits_{i=1}^S{\left({y}_i-\overline{y}\right)}^2{\sum}_{i=1}^S{\left({\hat{y}}_i-\overline{\hat{y}}\right)}^2} $$

2 $$ cvRMSE=\sqrt{\sum \limits_{i=1}^S\frac{{\left({y}_i-\hat{y_i}\right)}^2}{S}} $$

where, *y*_*i*_ is the measured activity of the *i*^th^ sequence, *ŷ*_*i*_ is the predicted activity of the *i*^th^ sequence, ȳ is the average and *S* the number of sequences. Apart from using them as evaluators of our predictions, they are also used to identify the most informative AAindex from a collection of 544 AAindices.

### Prediction schema

**Fig. 2:**
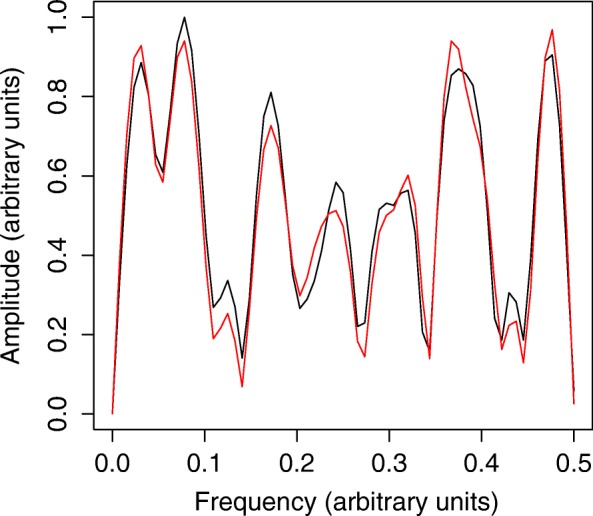
Fourier spectra of a protein sequence and a single point variant of the same protein. Shown are the Fourier spectra of wild type GLP-1 peptide (in blue) and of its E3A variant (in red). The spectra are obtained after numerically encoding the amino acid sequence using one index from AAindex database and their processing using Fast Fourier Transform (FFT) technique (see Methods section for details). A single point mutation impacts the whole spectrum. In the iSAR methodology, the variations caused by the mutation in the spectra of variants are correlated with variations observed in their corresponding biological activity using the PLS regression technique (see Fig. 3)

The amino acid sequences of variants are converted into a string of values corresponding to their physicochemical properties. To this end, 544 models are constructed corresponding to the 544 AAindices and the AAindex entry corresponding to the best performing model (highest R^2^ and the lowest cvRMSE) is chosen for numerical encoding. iSAR evaluates each of the encoding indices to find the best one for the construction of the model. For each index the set of sequences is encoded and a FFT is performed. iSAR uses the initial dataset (training set) to construct a predictive model for each encoding index. Then a cross-validation (leave-one-out or k-fold) for each computable component (ncomp) is performed by training a PLSR model. For each model, iSAR calculates the value of the performance parameters, cvRMSE and R2. The cvRMSE is the criterion used to calculate the coefficients in the PLS and to select the number of components of it. So, we use it also for the selection of the best encoding index: the final choice of the best index is therefore driven by the lowest value of cvRMSE. For each of the 4 datasets, we used the best index obtained through this procedure. PLS is used both to train the models and to find the best index. The mean of the numerical sequence is subtracted from itself. This procedure aims to cancel the first point of the spectrum at the zero frequency which is equal to the mean. Prior to the decomposition of the numerical signals using Fast Fourier Transform (FFT), the sequences have to satisfy the prerequisite that their length must be an exponent of 2 (2^n^). In our case, zeros are added at the end of the numerical sequences, to obtain a sequence length of 1024 (2^10^). This operation is called zero-padding. Then the Fast Fourier Transform (FFT) algorithm is run to transform the signal. The function “*fft”* implemented in R is used for this purpose (Eq. 3).

3 $$ {f}_j=\sum \limits_{k=0}^{N-1}{x}_k{e}^{\frac{-2 i\pi}{N} jk} $$

where, *j* is an index number of the Fourier transform, the numerical sequence includes *N* values denoted by *x*_*k*_ with 0 ≤ *k* ≤ *N*-1 and *N* ≥ 1 and *i* is the imaginary number such that *i*^*2*^ = −1. The module of the Fourier transform (|*f*_*i*_|) is computed in order to generate a *protein spectrum*. We use this *protein spectrum* (or spectral pattern of a protein) issued from digital signal processing as a descriptor to model the biological activity/fitness of protein from sequence data (Fig. 2).

Next, for the learning process, the aim is to set up a statistical model to link the fitness to the mutations. Using the *protein spectra* and the experimentally obtained fitness values, a PLS regression is performed. The R package “pls” is used for performing the regression [46]. For performing the PLS regression, the latent components are calculated as linear combinations of the original variables. The choice of the number of latent components to be considered for the PLS regression is based on the number of components that yield the least cvRMSE. The statistical model obtained by performing PLS regression on the training dataset is used to predict the fitness of the test dataset. The efficiency of the predictions is evaluated using the previously discussed statistical parameters R^2^ and cvRMSE. Both leave-one-out cross-validation (LOOCV), 10-fold cross-validation and 80–20 partitioning (80% training set and 20% test set) are performed on all the datasets. In LOOCV, except for the mutant for which the fitness is being predicted, the entire dataset is used for training; this process is repeated for all mutants. In 10-fold cross-validation the entire dataset is divided in 10 subsets containing each 1/10 mutants, 9 subsets are used for training, the tenth subset is predicted; this process is repeated for all subsets. In 80–20 cross-validation, the dataset is divided into 80% and 20% (by number of mutants) and the 80% is used for training and the remaining 20% for testing. Sequences in the test set were randomly selected using a random sampling procedure which preserves the class distribution of the result. A balanced split 80/20% of the data is run. The random sampling occurs within each class and should preserve the overall class distribution of the data. Using such procedure, when specifying 80–20%, it can be observed that the numbers of sequences in the test set does not fit exactly with 20% of the initial numbers of sequences, particularly when the number of sequences is low. For comparison with previous research works on cytochrome P450, a prediction is performed on the whole training set to determine the efficiency of the model thus built using a 10-fold cross-validation scheme.

**Fig. 3:**
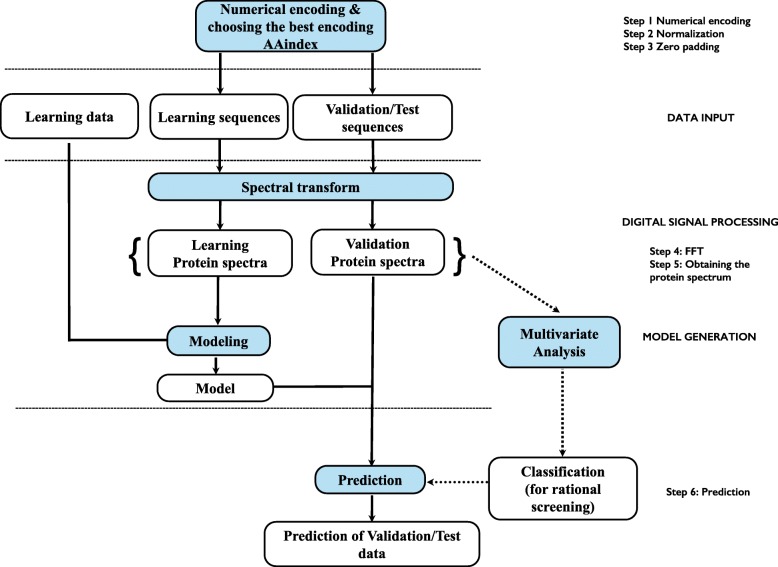
General scheme for the iSAR methodology described in this paper. “Multivariate Analysis” and “Classification (for rational screening)” on the right part of the figure are optional

Figure 3 is a schematic representation of the entire workflow for making the predictions. It should be noted that the blocks “Multivariate Analysis” and “Classification (for rational screening)” on the right part of Fig. 3 are optional and have not been used to obtain the results presented in this paper. The first part between the block “Validation – Protein spectra” and the block “Classification (for rational screening)” through the block “Multivariate Analysis” means that the protein spectra could be used directly for classification. The dotted lines from the block “Classification (for rational screening)” to the block “Prediction” means that this block could be optionally activated during the modelling and for the prediction. Using multivariate analysis such as Factorial Discriminant analysis, Principal Component Analysis, Random Forest… a classification of protein sequences according to their respective protein spectra could be performed. The main idea behind is that, if for example 3 classes are identified, a predictive model could be built for each of these classes in order to get a more specific model and better prediction of the fitness. When a new sequence has to be predicted, the prerequisite is that this new sequence is assigned to a class based only on its sequence information.

## Results

### Quantitative evaluation of the iSAR method

**Fig. 4:**
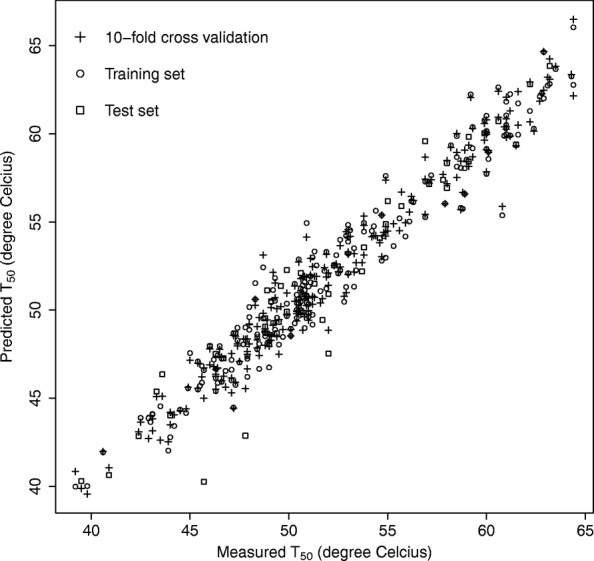
Evaluation of iSAR for modelling the thermostability of cytochrome P450 variants. Shown are the measured against predicted thermostability values (melting temperature in °C) assessed under the 10-fold cross-validation scheme for the full set of 242 ϖαριαντσ (+), for a training set composed of 80% of the dataset (○) and for a validation set comprising 20% of the variants (□)

Evidences that the relationship between sequence and activity/fitness can be modelled using iSAR in an efficient way are given through four different examples. The results for the evaluation of the predictions in terms of cvR^2^ and cvRMSE are summarised in Table 2. The comparison of the correlations between the predicted and measured activities for the cytochrome P450 dataset in 10-fold and 80–20 cross-validations are depicted in Fig. 4. Plots using LOOCV for the other three datasets are featured in Additional file 2: Figures S1-S3. The high values for R^2^ and low values for RMSE suggest that in every case, sequence information linked to the fitness was captured using our approach based on protein spectrum. Also, the fact that the LOOCV R^2^ and RMSE values for the enterotoxins dataset are as good as for the cytochrome P450 dataset indicates that a limited size of the dataset does not adversely affect the iSAR prediction abilities. Even by training only on 11 mutated sequences, iSAR was efficient in capturing the effect of mutations on thermostability.

**Table 2:** Summary of the different R^2^ and RMSE values obtained through predictions for the full set of protein sequences and after an 80/20 splitting in order to generate a training set and a validation set

Set	Partition	cvR^2^	cvRMSE
Cyt P450 (thermostability)	Full set (10-fold CV)	0.96	1.19
	Train set (80%) (10-fold CV)	0.93	1.33
	Validation set (20%)	0.92	1.72
Enterotoxins (thermostability)	Full set (LOOCV)	0.95	1.58
	Train set (80%) (LOOCV)	0.85	2.58
	Validation set (20%)	0.99	0.59
TNF (relative binding affinities)	Full set (LOOCV)	0.85	0.31
	Train set (80%) (LOOCV)	0.86	0.33
	Validation set (20%)	0.92	0.20
GLP-2 Potency (fold-increase in cAMP)	Full set (LOOCV)	0.42	2.05
	Train set (80%) (LOOCV)	0.75	1.39
	Validation set (20%)	0.71	1.44

For the full set and train set (80%), cvR^2^ and cvRMSE (same units as the activity for RMSE) values were evaluated after leave-one-out cross-validation (LOOCV) or 10-fold cross-validation scheme

### Modelling of unlearned mutations

Interestingly, for the GLP-2 validation set (R^2^ = 0.71), all the four randomly chosen variants had mutations at positions not sampled in the training set. Likewise, for the enterotoxins validation set (R^2^ = 0.99), two of the four sequences also had novel mutations: one sequence with seven new mutation positions and other with one new mutation position. The results for these two datasets are encouraging in the sense that the algorithm is demonstrating its capability to predict new mutations at novel positions that were not in the learning datasets. But we have to keep in mind that these values are averages over the entire dataset and individual mutants have to be analysed on a case by case basis.

It can be noted that for the GLP-2 validation set the R2 value is significantly higher than the R2 value obtained when a LOOCV is applied on the entire dataset: 0.71 and 0.42 respectively. This indicates the model is highly sensitive to the training set. Indeed, these specific 80% Train set and 20% validation set selected randomly give a high R^2^, but if another 80–20 partitioning would have arisen randomly, the R^2^ could be lower. Intuitively, we understand that if we try all the possible 80–20 partitions, the R^2^ should converge to a value close to the one obtained when a LOOCV procedure is run on the entire dataset i.e. close to 0.42. We did the experiment to exemplify this statement and indeed, if we use a k-fold =8 (so as to get a train set with 27 sequences and a validation set with 4 sequences) the R^2^ is 0.49, if we repeat this k-fold 100 times R^2^ is 0.41.

### iSAR is versatile with respect to the type of activity/fitness

Different types of biological activities or properties were modelled using our iSAR algorithm. For the TNF dataset, it was possible to model the preferential binding with one type of receptor (ratio R1/R2). The results for the GLP-2 indicates that the method performs also rather well for receptor activation (potency) as measured by experimental fold-increase in cAMP values. For enterotoxins and cytochrome P450, our results showed that the method was also able to model efficiently the thermostability of their variants. This versatility is explained by the fact that the algorithm finds the best encoding scheme that provides the best prediction accuracy (see below).

### Optimised numerical encoding scheme

The central dogma of protein biology is the strong interdependence between the sequence, structure and function of proteins. Since the iSAR methodology extrapolates the function from sequence, eliminating the need for the protein structure in between, the numerical encoding that captures this information further modelled by digital signal processing becomes an important step. The encoding based on the AAindices is more informative than the binary encoding method adopted by ProSAR.

The best encoding AAindex for the cytochrome P450 dataset is the D *Localized electrical effect* [47], while for the enterotoxins dataset it was the *Normalized frequency of isolated helix* [48] index. The best encoding scheme for modelling the relative binding affinity of TNF was the *AA composition of CYT2 of single-spanning proteins* [49]. As for the GLP-2 dataset, it was the *Hydropathy scale based on self-information values in the two-state model (20% accessibility)* [50] that best modelled the receptor activation. The fact that there are no two cases with the same AAindex indicates that different AAindices could be informative for different datasets: the best encoding could be determined for each couple sequences/activity on a case by case basis.

The Additional file 1: Table S5, sums up the protein features linked to the index found as the best one for each dataset. For each set, we used the best index from the selection of iSAR. Features linked to the index are: hydrophobicity (cytochrome P450), alpha and turn propensities (enterotoxin, GLP-2), average composition of amino acid composition of cytoplasmic region of transmembrane protein (TNF-alpha) and solvent accessibility of amino acid residues (GLP-2). It should be noted that, the selection of the Aaindex by the iSAR is done in a statistical approach. iSAR selects an index without the comprehension of the protein feature associated at this index.

Consequently:

- iSAR can associate a biological activity and one or several protein features without the existence of an obvious biochemistry link between the activity and the protein features.
- For different datasets with the same biological activity but different sequences, iSAR can select different indices and different protein features, like the case for the cytochrome P450 set and enterotoxin datasets where the fitness is a temperature.
- It is possible to have two indices selected by iSAR as the 2 best indices, and these indices are linked to two different protein features that does not have evident biochemistry links between them.

Furthermore, it is a strong assumption that only one physicochemical property governs the overall impact of the mutations on the protein’s activity. Therefore, for the GLP-2 dataset, for which the model has lower predictive ability, we have increased the number of physicochemical properties, by increasing the number of index, to try to improve the predictive ability of the model. Preliminary results show that when the 2 best indices are cumulated (Hydropathy scale based on self-information values in the two-state model (20%) [50] + Information measure for coil [51]), the cvR^2^ are respectively 0.43, 0.53 and 0.71 and cvRMSE 2.03, 1.90 and 1.44 for the full dataset, the 80% Train set and the 20% validation set. We can observe, in this case, a slight improvement for cvR^2^ (from 0.42 to 0.43) and cvRMSE (from 2.05 to 2.03) for the full dataset. Similar results are observed for the validation set. Even if the *p*-values associated to the calculation of cvR^2^ for the respective datasets (*p*-value = 8.35E-05, *p*-value = 6.08E-05) allow to state that for each model the predicted values are correlated to the measured ones, the Student’s t-test does not allow to conclude that the difference for the quadratic errors between the two models is significant (*p*-value = 0.92). Nevertheless, other combinations of index should be tested.

Currently, we are investigating to better understand why an index, or a combination of indices, is useful for the prediction of a target biological activity for a specific set of sequences.

## Discussion

### Comparison with other methods

The current method that we propose addresses some of the limitations of the method developed by Fox et al. [29, 52]: iSAR namely takes into account the effect of interactions between the residues at variable positions and the invariant residues. iSAR achieves this by considering the effect of the mutation on the fitness as a global phenomenon as opposed to a local phenomenon as considered by other methods implementing protein sequence to activity relationship (ProSAR). iSAR is also not limited by the ability to only predict fitness of mutation positions already explored in the training dataset. Here we demonstrated that it could predict mutations at new positions never learned in the training set.

For the thermostability of the cytochrome P450 dataset we can compare the results (using a 10-fold cross-validation scheme) with those obtained from Gaussian process models [53]: the R^2^ was 0.90, and with ProSAR using the PLSR-GA approach [31] R^2^ was equal to 0.94 and a RMSE equal to 1.52. In the iSAR approach we get an R^2^ of 0.96 and RSME of 1.19 using the same learning sequences. So, results obtained by this new method are better than those obtained using ProSAR for this cytochrome P450 example. Moreover, using the PLSR-GA approach of [52] results were obtained at the cost of integration of 45 interactions terms and much higher calculation time and computing power [31].

As seen in the *optimised numerical encoding scheme* section above, we have shown that the numerical encoding scheme of iSAR is more informative to effectively bring to light correlation that are not apparent otherwise. Our assumption that the effect of a single point mutation on the protein fitness is not purely local, but globally distributed over the linear sequence of the protein is corroborated in Fig. 2. We see that a single point mutation to the GLP-1 indeed impacts its entire *protein spectrum*.

Apart from these advantages, this method is also computationally less demanding, hence eliminating the need for protein engineers to handle specialised computational resources.

### Parameters affecting the performance of iSAR

The performance of iSAR depends on various factors namely the additivity of the mutations, quantitative and qualitative aspects of the experimental datasets and the sequence space to be searched. The additivity of the fitness upon mutations is the most important factor affecting the quality of the model. The more additive is the fitness upon mutations, the more the combinations of mutations will be predictable and thus lesser number of sequences will be needed in the training dataset. The preliminary requirement for the use of this approach is the availability of experimental data obtained through directed evolution experiments. The more these measurements are precise, accurate and reproducible, the better the method will be able to perform with a smaller number of learning sequences.

The sequence search space is another parameter that affects iSAR performance. The more the positions are mutated, the more sequences will be needed to capture the relative effect of each mutation. In this paper we have gotten nice results using small training sets as for enterotoxins or GLP-2. But it is obvious from a statistical point of view that the larger the experimental dataset, the better the prediction model will be.

One may therefore ask if the Fourier transformation into protein spectrum makes a significant improvement to the predictive ability of the models. If we consider the smallest (TNF) and the biggest full datasets (cytochrome P450), using a LOOCV procedure, the cvR^2^ drops from 0.85 with FFT to 0.64 without FFT and cvRMSE raises from 0.31 to 0.48. So, the Fourier transformation has a significant effect on the quality of the model. We are currently further deciphering the reasons for these observations. For cytochrome P450 it is simply not possible to run without FFT as the sequences vary from 464 to 466 residues. In the present case, zeros were added at the end of the numerical sequences to obtain a numerical vector of length 1024 (2^10^) in order to run the Fast Fourier Transform (FFT) algorithm. Indeed, this accelerates the FFT algorithm [54] and allows, in the case where the sequences are of different lengths, to have protein spectra of identical lengths.

The computational time for the predictions varies based on the size of the learning and test datasets. iSAR takes less than 2 min for a learning set of 242 sequences on a single CPU. It has been shown that the computing time may be the major limiting factor for the PLSR-GA approach [52], especially when the number of interaction terms to take into account is high [31].

## Conclusions

In this work, we have shown that the frequential representation (protein spectra) after Fourier transform can be used as descriptors in an efficient way in order to predict the protein activity of an amino acids sequence: the sequence-activity relationship can be modelled using the protein spectra. An important advantage of using frequential variables is that the comparison of amino acids sequences of different length becomes possible. Moreover, the frequential representation takes into account the impact of mutations on the whole spectral and does not focus on local. Our examples showed that in some cases, small learning datasets can be used to achieve good predictions and to obtain mutants with improved fitness.

## Additional files

Additional file 1: List of variants and corresponding activities for all 4 datasets used in this study. **Table S1.** GLP-2 variants with their measured and predicted activation. **Table S2.** Enterotoxin variants with their measured and predicted thermostabilities (in °C). **Table S3.** TNF alpha variants with their measured and predicted affinity. **Table S4.** Cytochrome P450 variants with their measured and predicted thermostabilities (in °C). **Table S5.** Summary of the protein features linked to the index found as the best one for each dataset. (PDF 342 kb)

Additional file 2: Evaluation of the iSAR methodology on several datasets. **Figure S1.** Plot of measured affinity of TNF variants versus predicted activity using iSAR algorithm. **Figure S2.** Plot of measured thermostability of enterotoxin variants versus predicted thermostability using iSAR algorithm. **Figure S3.** Plot of measured GLP-2R receptor activation of GLP-2 variants versus predicted receptor activation using iSAR algorithm. (PDF 875 kb)

## Abbreviations

cAMP: Cyclic adenosine monophosphate
CPU: Central processing unit
CRISPR/Cas9: Clustered Regular Interspaced Short Palindromic Repeats/CRISPR associated protein 9
cvR^2^: Coefficient of determination in Cross-Validation
cvRMSE: Root Mean Squared Error in Cross-Validation
DFT: Discrete Fourier Transform
EIIP: Electron-ion interaction potential
FFT: Fast Fourier Transform
GLP: Glucagon like peptide
HLA: Human leukocyte antigen
iSAR: Innovative Sequence Activity Relationship
LOOCV: Leave-one-out cross-validation
MSH: Melanocyte-stimulating hormones
PLS: Partial Least Squares
PLSR-GA: Genetic algorithm (GA) combined with partial least squares (PLS) regression
ProSAR: Protein Sequence Activity Relationship
QSAR: Quantitative structure-activity relationship
QSFR: Quantitative Structure Function Relationship
QSSR: Quantitative Structure Stability Relationship
R^2^: Coefficient of determination
RMSE: Root Mean Squared Error
RRM: Resonant Recognition Model
SEA: Staphylococcal enterotoxins A
SEE: Staphylococcal enterotoxins E
TNF: Tumour Necrosis Factor
TNFR: Tumour Necrosis Factor Receptor
WT: Wild Type
