# Supplementary material for: Application of fourier transform and proteochemometrics principles to protein engineering
Source: BMC Bioinformatics. 2018 Oct 16;19:382. doi: 10.1186/s12859-018-2407-8 (PMC6191906; doi:10.1186/s12859-018-2407-8)
Supplement: Supplementary file 1 — List of variants and corresponding activities for all 4 datasets used in this study. Table S1. GLP-2 variants with their measured and predicted activation. Table S2. Enterotoxin variants with their measured and predicted thermostabilities (in °C). Table S3. TNF alpha variants with their measured and predicted affinity. Table S4. Cytochrome P450 variants with their measured and predicted thermostabilities (in °C). Table S5. Summary of the protein features linked to the index found as the best one for each dataset. (PDF 342 kb) [file 12859_2018_2407_MOESM1_ESM.pdf]

Application of Fourier Transform and proteochemometrics principles to protein engineering

Additional file 1

List of variants and corresponding activities for all 4 datasets used in this study.

Table S1. GLP2 variants with their measured and predicted activation (see Methods for details about the measured activation). Variants in red were used in the test set.

Variant	Measured	Predicted
A2G	10.00	5.53
H1A	0.78	1.37
D3A	1.02	2.38
G4A	2.87	1.11
**S5A**	4.00	1.59
F6A	1.67	5.13
S7A	7.59	6.19
D8A	7.53	3.80
E9A	3.76	5.12
M10A	0.78	2.75
N11A	7.65	8.50
T12A	3.11	1.75
I13A	2.45	4.93
L14A	2.03	3.10
D15A	4.54	4.43
**N16A**	5.92	1.94
L17A	1.02	2.42
R20A	3.94	5.35
D21A	4.42	0.67
F22A	1.61	1.79
I23A	2.57	1.08
N24A	10.00	10.05
W25A	1.37	0.94
**L26A**	2.33	4.13
I27A	5.02	4.36
Q28A	5.80	7.06
T29A	4.78	2.63
K30A	0.84	-0.94
I31A	4.42	2.93
**T32A**	0.84	4.24
D33A	3.41	3.44

Table S2. Enterotoxin variants with their measured and predicted thermostabilities (in °C). Variants in red were used in the test set.

Variant	measured	predicted
**SEA_D227A**	55.1	55.8
SEA_H187A	57.5	58.5
SEA_233aa	61.4	58.5
SEA/E-bdeg	68.4	69.6
SEE/A-h	69	67.3
SEE/A-a_D227A	69.3	70.8
SEE_233aa	71.3	72.0
SEE/A-a	75.3	73.4
**SEE_A-f**	70	70.1
**SEE_A-ah**	69.1	71.1
**SEE_D227A**	67.4	66.9
SEA_D227A_F47A	55.4	55.5

Table S3. TNF alpha variants with their measured and predicted affinity (see Methods for details about the measured affinity). Values are normalized with respect to WT. Variants in red were used in the test set.

Variant	measured	predicted
WT_157aa	0	0.060
K11M_K65S_K90P_K98R_K112N_K128P	0.079	0.100
L29I	0.079	0.469
A84T_V85H_S86K_Q88P_T89Q	0.544	0.501
**A84S_V85K_S86T_Q88S_T89H**	0.663	0.763
**L29Q_R32W**	0.826	1.124
L29K_R31A_R32G_E146S_S147T	0.924	1.413
A84S_V85T_S86N_Q88N_T89G	0.869	1.231
A84S_V85S_S86H_Q88R_T89F	1.079	1.003
A84S_V85P_S86L_Q88P_T89K	1.217	0.730
**A84T_V85S_S86A_Q88G_T89P**	1.23	1.586
A84T_V85T_S86A_Q88S_T89G	1.31	1.008
A145R_E146T_S147D	1.301	1.450
A145K_E146D_S147T	2.87	2.935
A145R_E146E_S147T	2.228	2.018
A145A_E146D_S147D	1.949	2.311
A145A_E146N_S147D	2.462	2.051
L29T_R31G_R32Y	0.38	0.331
L29T_R31K_R32Y	1.127	0.903
**L29T_R32F_E146T**	2.026	2.853
A84S_V85K_S86T_Q88T_T89H	0.924	0.932

Table S4. Cytochrome P450 variants with their measured and predicted thermostabilities (in °C). Variants are chimeric constructs of the enzyme as detailed in Romero et al, 2013[1]. Variants in red were used in the test set.

Variant	measured	predicted	Variant	measured	predicted	Variant	measured	predicted
11111111	55.0	54.5	22133232	47.9	46.7	21132222	45.6	46.2
22222222	43.0	44.0	22233221	46.8	45.6	21212333	63.2	63.1
33333333	49.0	47.9	23113323	51.0	49.4	**21231233**	50.6	51.8
32233232	39.8	39.6	**11332212**	47.8	45.5	22212322	50.7	51.0
32313233	52.9	54.5	**32332231**	49.4	48.5	**21112122**	50.3	50.5
21133233	48.8	48.9	22132331	53.3	52.2	22111223	51.3	51.9
31312113	45.0	47.2	**23313111**	56.9	55.4	23233212	39.5	39.9
21332223	48.3	50.6	23112323	46.0	47.8	31312212	48.9	49.4
21312323	61.5	59.3	11113311	51.2	50.8	32211323	46.6	46.0
22312322	54.6	53.8	21232233	50.6	51.1	21213231	54.9	57.4
21212112	51.2	52.5	12332233	47.1	46.2	21332312	52.9	51.0
23133121	47.3	45.9	**23333311**	45.7	46.7	22332211	53.0	52.0
11312233	51.6	50.4	32132233	42.9	42.7	22113323	53.8	55.3
21133312	45.4	47.0	22331123	47.9	47.5	22113332	48.7	53.1
21133313	50.8	51.9	12212332	48.4	47.8	22213132	52.0	48.9
11332233	43.3	45.1	31212323	48.7	49.8	31213332	50.8	49.6
31212332	53.4	52.7	32312322	49.1	50.1	**22113211**	51.1	52.0
12211232	49.1	48.3	32312231	52.6	53.6	22313323	60.0	59.1
31312133	52.6	52.4	21232332	49.3	50.7	32333233	47.2	48.6
12232332	39.2	40.8	**31331331**	47.3	48.5	22331223	51.7	50.6

[1] Romero, P. A., Krause, A., & Arnold, F. H. (2013). Navigating the protein fitness landscape with Gaussian processes. *Proceedings of the National Academy of Sciences of the United States of America*, 110(3), E193–201. http://doi.org/10.1073/pnas.1215251110

Table S4 (contd).

Variant	measured	predicted	Variant	measured	predicted	Variant	measured	predicted
**23333233**	51.0	50.0	22232233	49.6	50.2	21113322	50.4	49.4
22333332	49.0	48.3	22232322	45.4	45.6	**31313232**	51.9	51.9
23332331	48.0	49.2	22333211	50.7	51.9	23213333	56.1	55.6
21233132	42.4	43.1	22332223	52.4	52.6	21333233	54.2	54.2
**13333211**	45.7	45.0	23213212	49.0	48.6	22233212	44.0	43.5
22232331	50.5	50.0	23333213	50.1	48.5	21313112	54.8	54.4
22313233	58.5	60.0	31312233	57.9	56.0	31213233	50.6	49.7
31311233	56.9	57.4	22232333	53.7	52.7	22132113	40.6	42.0
21132321	49.3	49.4	31333233	46.5	47.8	31112333	55.7	56.7
32212231	47.4	48.1	**22213212**	50.5	50.3	31212331	51.8	52.4
23212212	48.0	49.2	**22132212**	46.6	47.3	22232222	47.5	47.1
22113223	49.9	49.7	21332233	58.9	56.6	**23332221**	46.4	46.7
22233211	46.3	46.4	23333131	50.5	52.7	21332131	58.5	59.0
**23213311**	49.5	47.5	31312332	54.9	54.7	23231233	45.5	46.9
31212321	44.9	45.6	21333221	51.3	50.7	**22111332**	50.9	50.8
23112233	51.0	50.7	22333223	49.9	51.0	23312121	49.3	51.5
**32332323**	48.5	47.4	**21111333**	62.4	60.1	**22332222**	50.3	51.4
22112223	52.8	50.8	12212212	44.8	44.4	23312323	53.8	53.1
32313231	52.5	52.2	11313233	48.3	50.3	**21131121**	53.0	54.1
32132232	42.5	43.6	**32113232**	47.9	48.4	32212232	48.8	48.8

Variant	measured	predicted	Variant	measured	predicted	Variant	measured	predicted
22112323	55.3	54.9	23313333	61.2	59.9	32212323	48.4	49.1
**21232232**	49.5	49.5	21113133	51.9	53.2	21212111	57.2	57.4
11212333	50.4	49.8	21111323	54.4	54.2	31212212	47.1	45.3
**31212232**	51.0	49.8	22212123	47.7	48.4	**22232121**	49.7	48.9
**23213211**	47.4	48.0	12211333	50.6	50.8	21232212	47.8	48.3
11331312	43.5	42.6	23113112	46.3	46.6	21333223	49.1	48.5
23331233	50.9	51.9	21313122	50.5	50.6	23213232	48.5	49.6
22133323	49.4	50.1	23112333	54.3	54.2	22113232	51.1	52.9
33333233	46.3	45.5	12213212	44.0	44.2	11331333	46.3	46.2
22233323	48.4	48.3	23132233	43.6	45.1	22333321	49.2	49.1
**32232131**	43.9	42.5	**21313311**	56.9	58.7	21232321	46.0	46.9
31312323	52.3	52.5	**21332231**	60.0	57.8	**31332233**	49.9	49.7
21313313	64.4	62.2	23133233	43.1	43.2	21133232	46.4	46.7
22333231	53.1	54.2	21132212	48.8	48.7	22112211	54.7	53.0
22232123	43.1	43.8	23313233	56.3	56.2	21333333	58.0	57.2
21132323	50.1	48.9	21332322	48.8	48.0	**22213223**	50.8	50.6
23332231	51.4	51.9	22132231	53.0	53.2	21332112	50.4	49.9
12112333	50.9	50.3	**21113312**	53.0	53.3	**21331332**	52.0	52.1
**22133212**	47.2	44.4	**22312223**	56.2	56.4	**11313333**	53.8	54.8
31113131	54.9	54.2	23332223	46.7	46.3	**32311323**	52.0	51.4

Variant	measured	predicted	Variant	measured	predicted	Variant	measured	predicted
23132231	48.0	48.1	21313231	61.0	60.3	21312311	59.1	59.1
12232232	40.9	41.1	22312133	57.1	57.2	22313333	64.3	62.3
21212231	59.9	59.6	22312231	60.0	60.8	21311313	61.2	60.9
33312333	54.7	55.4	22312311	55.6	54.5	21312213	60.6	60.9
22313232	58.8	56.7	**22312332**	59.1	58.1	21312332	59.9	60.6
22312111	53.0	54.6	**22312333**	63.5	63.8	**21311231**	63.2	63.6
32212233	49.9	49.8	21312333	64.4	66.5	22312313	61.0	58.8
21132112	47.1	47.5	**12322333**	47.9	47.6	22311331	58.9	58.7
**23132311**	44.5	44.3	21312331	60.6	62.4	21312133	60.1	60.8
21312123	60.8	55.9	21311333	59.2	62.1	22311233	60.9	61.0
23133311	44.2	44.1	21312233	63.1	63.2	21311311	61.0	61.4
22113111	49.2	52.1	21313333	62.9	64.7	22313331	58.5	57.9
23212211	50.7	48.9	21312313	62.2	62.9	**21312211**	59.3	61.2
21212321	53.3	53.2	**21312231**	62.8	62.3	21112333	61.6	61.6
**21333211**	55.9	54.9	21311233	62.7	61.9	22313231	59.0	58.5
22232212	46.2	46.8	21313331	62.2	60.7	21212233	60.0	58.9
23313323	50.9	54.1	22312331	59.3	58.7	21112331	61.6	59.6
**32312333**	57.8	57.7	**22311333**	60.1	59.1	21112233	58.7	58.3
12313331	51.2	50.3	22312233	61.0	62.1	**22212333**	58.2	58.7
21311331	62.9	62.5	21313233	60.0	60.1	22112333	58.0	59.1
						21113333	61	60.7
						22112233	58.7	55.9

Table S5. Summary of the protein features linked to the index found as the best one for each dataset.

Set	Activity	Index	Protein features linked to the index
P450	thermostability	Localized electrical effect	Hydrophobicity
enterotoxin	thermostability	Normalized frequency of isolated helix	alpha and turn propensities
TNF-alpha	relative binding affinities	AA composition of CYT2 of single-spanning proteins	Composition of amino acid
GLP2	Potency	Hydropathy scale based on self-information values in the two-state model (20% accessibility)	Solvent accessibility of amino acid residues
GLP2	Potency	Information measure for coil	alpha and turn propensities
