# Supplementary material for: Application of fourier transform and proteochemometrics principles to protein engineering
Source: BMC Bioinformatics. 2018 Oct 16;19:382. doi: 10.1186/s12859-018-2407-8 (PMC6191906; doi:10.1186/s12859-018-2407-8)
Supplement: Supplementary file 2 — Evaluation of the iSAR methodology on several datasets. Figure S1. Plot of measured affinity of TNF variants versus predicted activity using iSAR algorithm. Figure S2. Plot of measured thermostability of enterotoxin variants versus predicted thermostability using iSAR algorithm. Figure S3. Plot of measured GLP-2R receptor activation of GLP-2 variants versus predicted receptor activation using iSAR algorithm. (PDF 875 kb) [file 12859_2018_2407_MOESM2_ESM.pdf]

Application of Fourier Transform and proteochemometrics principles to protein engineering

Additional file 2

Evaluation of the iSAR methodology on several datasets.

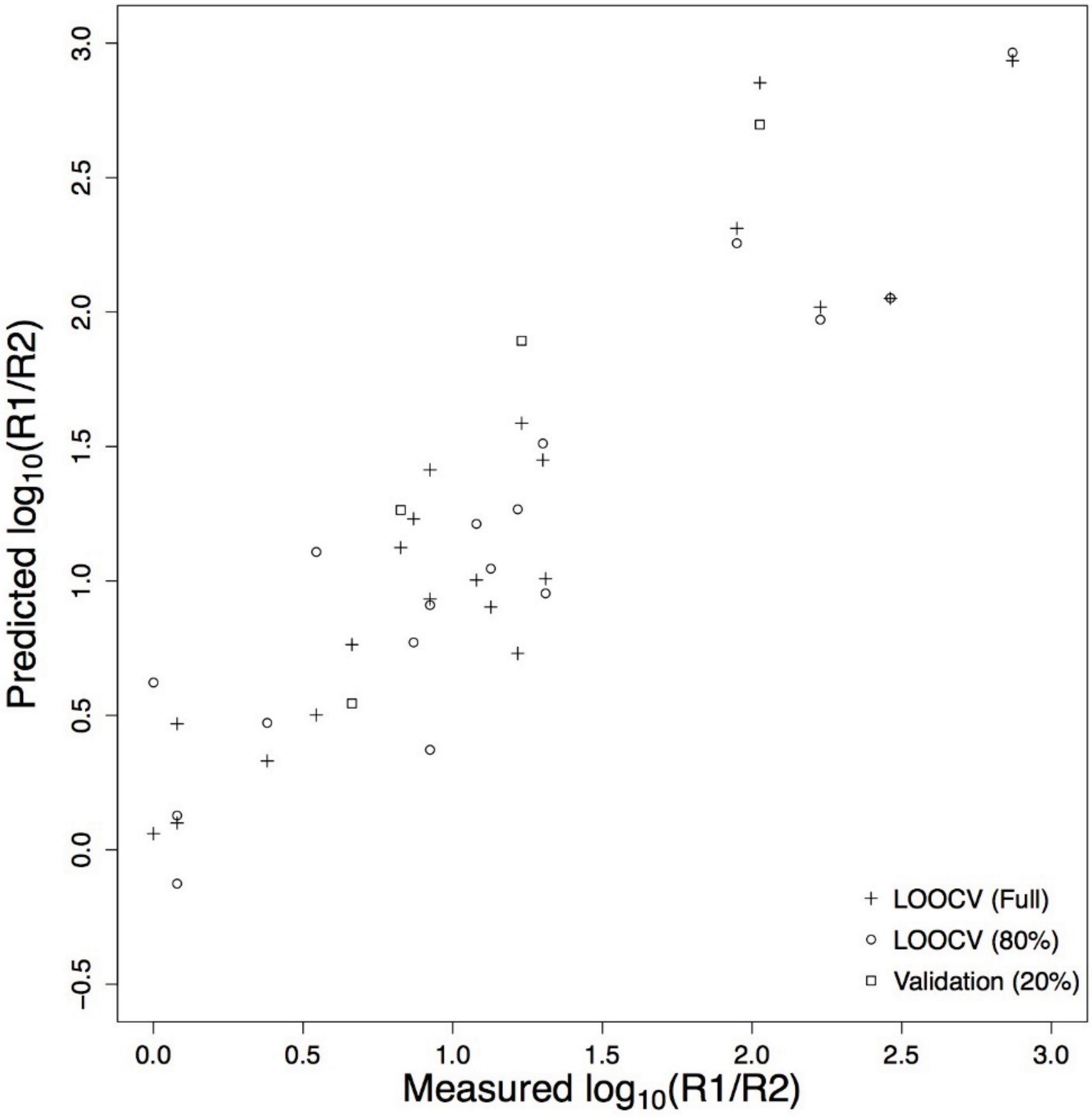

Figure S1. Plot of measured affinity of TNF variants versus predicted affinity using iSAR algorithm.

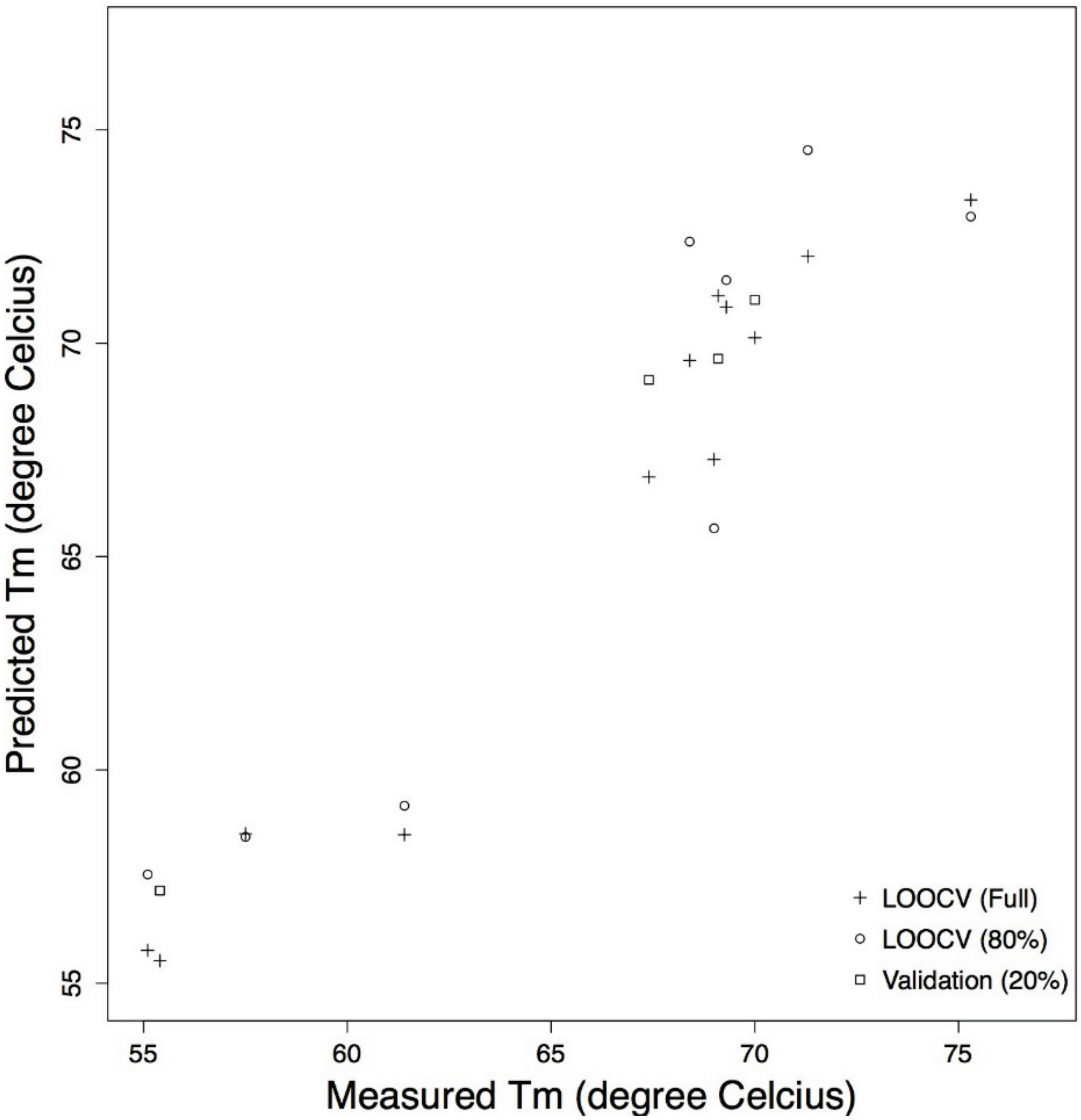

Figure S2. Plot of measured thermostability of enterotoxin variants versus predicted thermostability using iSAR algorithm.

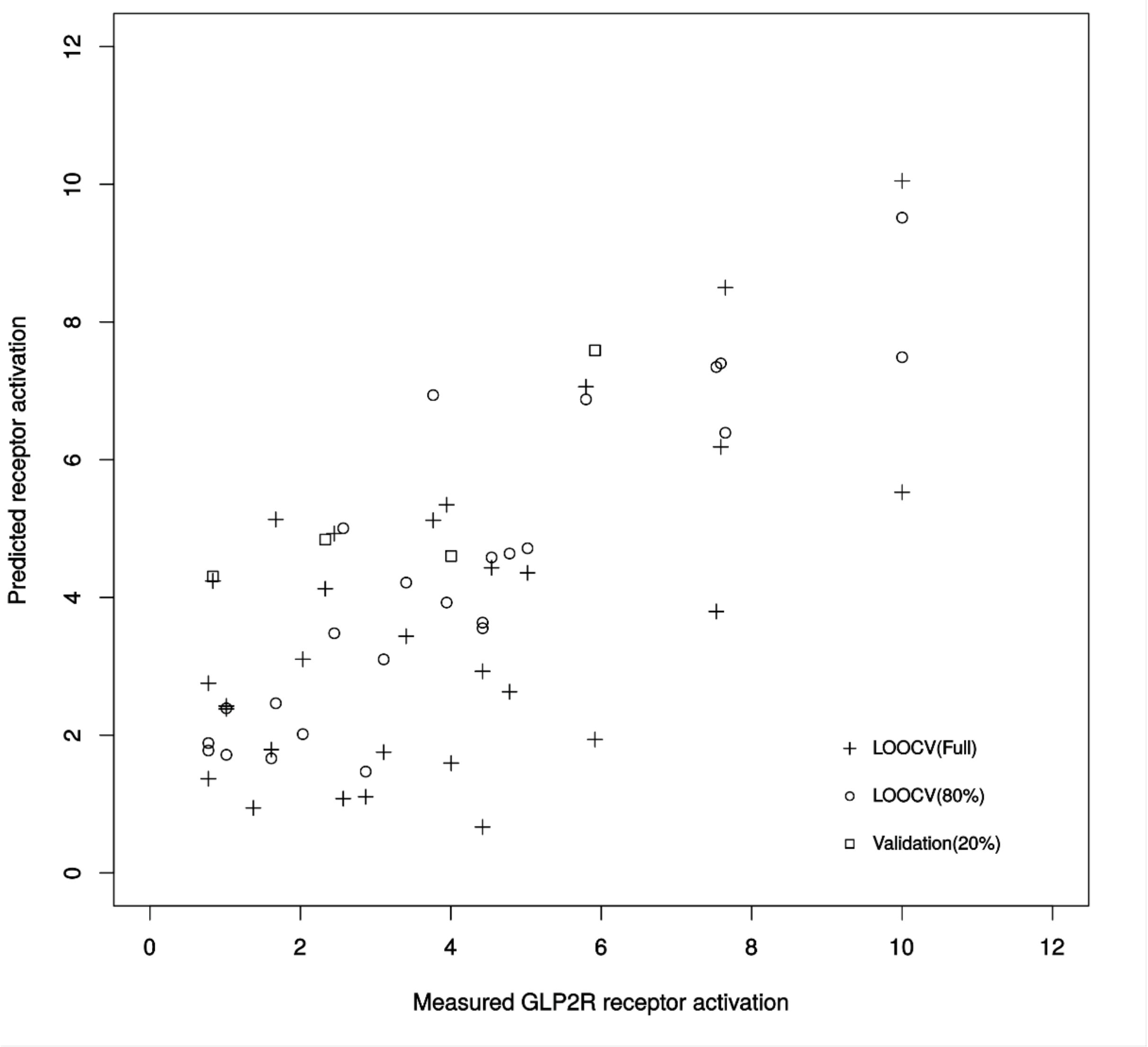

Figure S3. Plot of measured GLP2R receptor activation of GLP2 variants versus predicted receptor activation using iSAR algorithm
